# Simulation Study of Radio Frequency Safety and the Optimal Size of a Single-Channel Surface Radio Frequency Coil for Mice at 9.4 T Magnetic Resonance Imaging

**DOI:** 10.3390/s22114274

**Published:** 2022-06-03

**Authors:** Jeung-Hoon Seo, Yeunchul Ryu, Jun-Young Chung

**Affiliations:** 1 Neuroscience Research Institute, Gachon University, Incheon 21988, Korea; jeunghoon.seo@gachon.ac.kr; 2 Department of Radiological Science, Gachon University, Incheon 21936, Korea; 3 Department of Neuroscience, College of Medicine, Gachon University, Incheon 21565, Korea

**Keywords:** radiofrequency coil, finite-difference time-domain, preclinical magnetic resonance imaging, 9.4 T magnetic resonance imaging system, specific absorption rate, radiofrequency safety, ultra-high field magnetic resonance imaging

## Abstract

The optimized size of a single-channel surface radio frequency (RF) coil for mouse body images in a 9.4 T magnetic resonance imaging (MRI) system was determined via electromagnetic-field analysis of the signal depth according to the size of a single-channel coil. The single-channel surface RF coils used in electromagnetic field simulations were configured to operate in transmission/reception mode at a frequency of 9.4 T–400 MHz. Computational analysis using the finite-difference time-domain method was used to assess the single-channel surface RF coil by comparing single-channel surface RF coils of varying sizes in terms of |B_1_|-, |B_1_^+^|-, |B_1_^−^|- and |E|-field distribution. RF safety for the prevention of burn injuries to small animals was assessed using an analysis of the specific absorption rate. A single-channel surface RF coil with a 20 mm diameter provided optimal B_1_-field distribution and RF safety, thus confirming that single-channel surface RF coils with ≥25 mm diameter could not provide typical B_1_-field distribution. A single-channel surface RF coil with a 20 mm diameter for mouse body imaging at 9.4 T MRI was recommended to preserve the characteristics of single-channel surface RF coils, and ensured that RF signals were applied correctly to the target point within RF safety guidelines.

## 1. Introduction

Recently, preclinical researches of human diseases have been frequently studied by using small animal models of artificially generated human diseases such as tumors and neurodegeneration. The use of mice or rats enables significant cost savings, particularly for studies using expensive chemicals such as contrast mediums, even when factoring in the cost of managing animals. Therefore, the rodent has become a key animal in the development of model diseases [1,2,3,4,5,6,7,8,9,10,11]. Magnetic resonance imaging (MRI) is particularly suitable for animal model investigation because it provides functional, anatomical, and/or physiological information without affecting animal integrity. Furthermore, due to the high image quality produced by ultra-high field (UHF) MRI scanners, they have been used in a wide range of preclinical applications. As interest grows in the use of small rodents such as mice and rats in animal models for conducting researches on human diseases, there has emerged a new trend of utilizing high-resolution images produced with preclinical MRI scanners at UHF strengths (≥7.0 T) [12,13]. For this reason, preclinical researches in UHF MRI have been actively underway, and the importance of high-performance radiofrequency (RF) coils has emerged to obtain high-resolution anatomical images in preclinical MRI systems for small animals [14,15,16,17,18,19]. The researches to improve the performance of RF coils have been conducted in various ways in both preclinical MRI and clinical MRI fields, of which studies using additional structures (such as wireless element and HPM) [20,21,22,23,24,25,26,27,28,29,30,31,32,33,34,35], transmission line based RF coil to allow it to be independent of the length of the RF coil [36,37,38,39,40,41], and researches on the dedicated shapes of RF coils themselves have mainly focused [15,42,43,44,45,46,47,48]. In addition, research on volume coils such as birdcage coils at various magnetic field strengths [49] and the development of new RF coil techniques have been actively conducted [50].

Especially in preclinical magnetic resonance (MR) experiments, MR images are obtained by controlling animal movements with anesthesia. Preclinical studies are mainly conducted using single-channel surface RF coils, thus making it easy to install and capable of high signal sensitivity images that are close to the target; however, preclinical MR images must be acquired quickly before the animal awakens from anesthesia. Due to these limitations, single-channel surface RF coils have been designed for operation primarily in transmit/receive (Tx/Rx) configurations [24,51,52]. However, preclinical experiments have been conducted without considering the optimal size of RF coils depending on the main magnetic field strength and type of animal models. Therefore, it is necessary to configure a single-channel RF coil that provides sufficient signal depth for the size of the animal used in the preclinical experiment, as well as the distance to the target point for image acquisition.

In addition, preclinical MRI studies should be conducted in adherence to ethical guidelines for animal use [53,54,55,56,57,58,59]. In terms of animal ethics and morality of animal use in human society, this paper aimed to improve animal ethics by securing the minimum RF safety of experimental animals sacrificed for humans in MRI preclinical experiments of mice. In MRI experiments, the RF safety guideline that is relevant to the selection of RF coils is the risk of burn injuries, which may occur when the RF coil system is not configured properly for the animal models; it can be mitigated by considering the specific absorption rate (SAR) [60,61,62,63,64,65]. Although RF safety is generally considered when conducting clinical MRI studies, most preclinical MRI studies have relatively neglected the consideration of RF safety due to a lack of established ethical guidelines for the SAR in small animal models. Nevertheless, the physiological effects of tissue heating due to heavy RF deposition in small animals could be a serious confounding factor in preclinical MRI studies and should not be ignored. Because it may be difficult to obtain accurate MR images without considering the SAR, tissue changes caused by tissue heating may not protect the rights of animals. In MRI studies, most burn injuries occur due to SAR problems in RF coils, and this risk increases as magnetic field strength increases [66,67,68].

In this work, we proposed the optimal size of a single-channel surface RF coil that could provide sufficient signal depth when acquiring mouse body images using preclinical MRI at 9.4 T. To propose the optimal size and RF safety of a single-channel surface RF coil, electromagnetic field (EM-field) analysis was performed using finite-difference time-domain (FDTD) methods by adjusting the diameter of the single-channel coil from 30 mm to 10 mm with 5 mm intervals. The single-channel surface RF coils used in the EM-field simulation were 10 mm, 15 mm, 20 mm, 25 mm, and 30 mm in diameter. Three types of numerical phantoms for EM-field simulation were used: oil phantom, water phantom, and mouse phantom. The oil phantom and water phantom were used to compare the relative changes in signal depth under various dielectric properties to the size of a single-channel surface RF coil, whereas the mouse phantom was used to assume an actual MR experiment.

## 2. Materials and Methods

To evaluate the optimal size of single-channel surface RF coils for preclinical MRI, EM-field simulations were performed using Sim4Life™ v4.4 (Zurich MedTech AG, Zürich, Switzerland) commercial software, which is widely used in numerical calculations using the FDTD method based on Yee cells [69]. EM-field analysis was validated in terms of the signal depth of |B_1_|-field and SAR distribution due to the |E|-field concentration, depending on the size of the single-channel surface RF coil.

**Figure 1:**
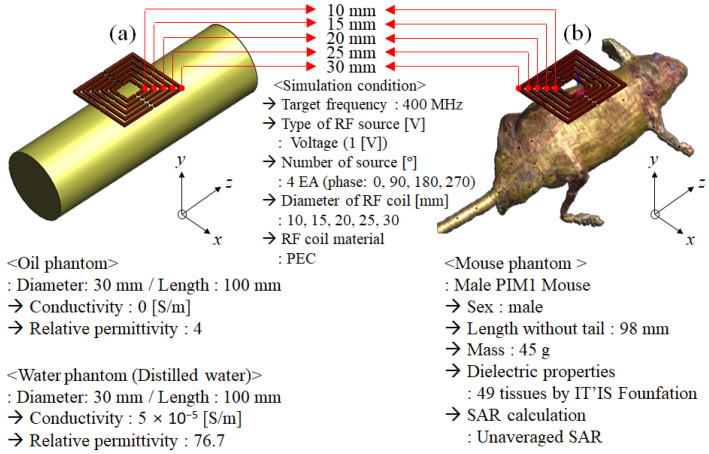
Geometric structures and simulation conditions of electromagnetic field simulation for the finite-different time-domain (FDTD) method using an oil phantom, water phantom, and mouse model: (**a**) oil phantom and water phantom with dielectric properties; (**b**) mouse phantom with dielectric properties.

In Figure 1, for EM-field simulation, the single-channel surface RF coils were constructed in a square shape using perfect electric conductor material. The diameter of single-channel surface RF coils was set at a 5 mm interval from 10 mm to 30 mm (10 mm, 15 mm, 20 mm, 25 mm, and 30 mm). The single-channel surface RF coil for the Tx/Rx mode had four ports as a voltage source of 1 V and the geometrical phase (0°, 90°, 180°, and 270°). The EM-field calculation was performed with a target frequency of 400 MHz for the 9.4 T MRI system. The target frequency (ω) was defined as the gyromagnetic ratio (γ) and main magnetic field strength ($B_{0})$ as following:

(1) $$\omega =\gamma B_{0}$$

The volumetric RF coil, since the RF transmission field was excited by the iso-center of the RF coil, achieved a B_1_^+^-field strength of 2.0 μT based on the center of the RF coil. However, it was difficult to quantitatively analyze the surface coil because various results were derived according to the change in the position of the target point when analyzing the field by randomly setting the target point. Therefore, in general, in the FDTD analysis of the surface coil, the RF power was fixed, and the analysis was performed in terms of signal depth and sensitivity of the RF coil.

The numerical phantoms for EM-field simulation utilized the cylindrical phantom (oil phantom and the water phantom (shown in Figure 1a)) and the mouse phantom (Male PIM1 Mouse by IT’IS Foundation (Information Technologies in Society), Switzerland), as shown in Figure 1b. The oil phantom and water phantom had a diameter of 30 mm and a length of 100 mm. The oil phantom consisted of dielectric properties with a conductivity of 0 S·m^−1^ and a permittivity of 4. Meanwhile, the water phantom using distilled water consisted of dielectric properties with a conductivity of 5 × 10^−5^ S·m^−1^ and a permittivity of 76.7. The reason for performing the simulation using the oil phantom was to evaluate the quantitative performance of the RF coil and to verify the EM-fields generated by the RF coil itself under ideal conditions. As the strength of the magnetic field increases, RF field inhomogeneity occurred by shifted |B_1_^+^|-field and |B_1_^−^|-field, it became difficult to quantitatively evaluate the unique characteristics of RF coils [70,71]. In particular, it was difficult to quantitatively evaluate the RF field due to the inhomogeneity of the |B_1_^+^|-field and |B_1_^−^|-field since the reciprocity theorem was not applied to ultra-high field MRI above 7.0 T, and this inhomogeneity became more severe as it has higher conductivity. For this reason, the EM-field generated by the RF coil could be quantitatively evaluated in simulation using the oil phantom, whereas the EM-field simulation using the water phantom, which consisted of distilled water, could calculate EM-fields similar to the MR images using the actual mice assuming that there was a ^1^H proton signal inside the water molecule of the experimental mice. The mouse phantom had a length of 98 mm without a tail and a mass of 45 g, with 49 tissue parameters. The PIM1 mouse phantom was based on MR segmented data, and each of the 49 defined tissue parameters was described analytically. The tissue parameters were assigned, such as electric conductivity, permittivity, and permeability for EM-field simulations. Tissue parameters for application to thermal simulations (such as thermal conductivity, heat generation rate, heat transfer rate, and heat capacity) were also defined in detail. The tissue parameter values, including density and dielectric properties, were included in the material database provided by IT’IS Foundation (DOI: 10.13099/VIP91201-01-0) [72].

The distance between the single-channel surface RF coil and each phantom (Oil phantom, water phantom, and mouse phantom) was set to be the closest possible distance of 1 mm. In clinical MRI, MR images could be obtained that provide higher sensitivity and uniformity by using a multi-channel RF coil configured with volumetric features in ultra-high field MRI. However, in the case of preclinical MRI, it was difficult to insert both volumetric multi-channel RF coils and experimental animals within the limited magnet bore size. Therefore, in order to obtain a higher SNR MR image, the surface coil was used mainly in contact with object as close as possible to obtain the image.

For numerical calculations, the computational space composed of Yee cells was set to 86 × 83 × 156 cells (1.114 mega cells) for oil phantom and 171 × 166 × 418 cells (11.865 mega cells) for mouse phantom along the *x*, *y*, and *z* directions. Along the *x*, *y*, and *z*-axis, three-dimensional Yee cells were adopted with a resolution of less than 1 mm, including the absorbing boundary condition with a perfectly matched layer for the acquisition of accurate EM-field distribution and −70 dB conversions to produce a steady-state equilibrium condition of a single-channel surface RF coil. EM-field simulations were calculated by a complex data matrix using MATLAB (Version 2020a, MathWorks, Inc., Natick, MA, USA). The EM-field simulation results calculated using MATLAB were displayed by setting the voxel resolution to 0.2 × 0.2 × 0.45 mm.

The single-channel surface RF coil produces a highly local EM-field sensitivity depending on the signal depth. The signal depth is defined as the point at which the sensitivity of the single-channel surface RF coil drops to 37% of that at the center of the single-channel surface RF coil. The signal depth of the single-channel surface RF coil is proportional to the diameter of the coil [73,74]. In other words, as the coil diameter increases, the interference with the target to obtain the MR image increases, which means that noise coming through the target tissue is affected. In addition, as the size of the single-channel surface RF coil decreases, a lot of RF power is required to acquire MR images in the target region. For this reason, signal depth optimization had to be performed to optimize the single-channel surface RF coil. In addition, RF safety optimization considered SAR should also be performed to ensure the safety of experimental mice.

To analyze the performance of the single-channel surface RF coil in terms of the signal depth and sensitivity, we compared the |B_1_|-, |B_1_^+^|-, and |B_1_^−^|-field distributions depending on the size of the single-channel surface RF coil at the center slice. |B_1_|-, |B_1_^+^|-, and |B_1_^−^|-fields refer to absolute values of B_1_, B_1_^+^, and B_1_^−^ fields. The B_1_ field distribution involving the *x*- and *y*-component as expressed as B_1*xy*_ as follows:

(2) $$B_{1}=\sqrt{{\left(B_{1x}^{+}\right)}^{2}+i{\left(B_{1y}^{-}\right)}^{2}}$$

And B_1_ includes two circularly polarized components defined as B_1_^+^ and B_1_^−^, where B_1*x*_ and B_1*y*_ are B_1_ components on the *x*-axis and *y*-axis, respectively. B_1_^+^ and B_1_^−^ can be defined as follows:

(3) $$B_{1}^{+}=\left|\frac{\left(B_{1x}+iB_{1y}\right)}{\sqrt{2}}\;\right|,{\;B}_{1}^{-}=\left|\frac{\left(B_{1x}-iB_{1y}\right)}{\sqrt{2}}\;\right|$$

**Figure 2:**
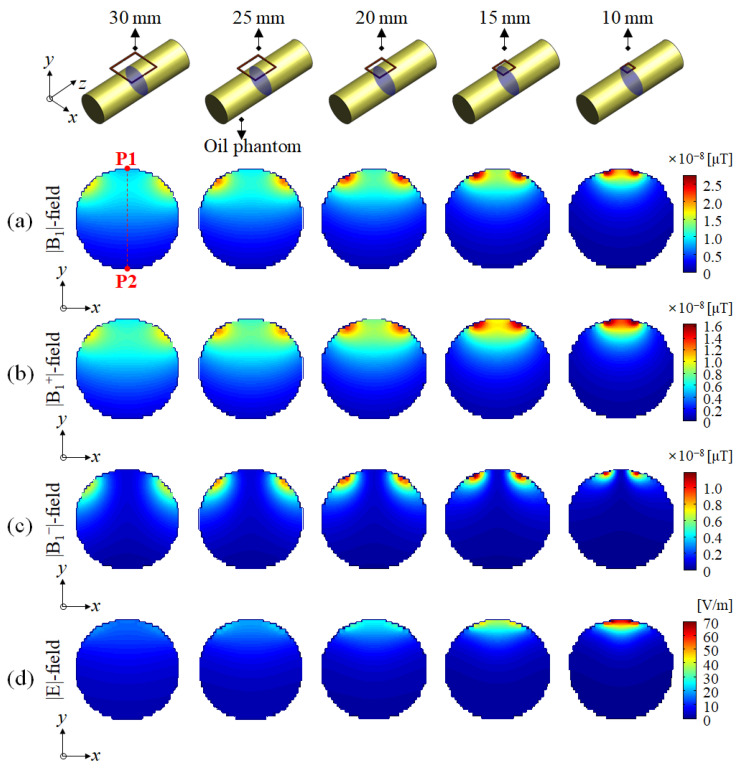
EM-field simulation results using oil phantom in the axial slice (*x*–*y* plane): (**a**) |B_1_|-field, (**b**) |B_1_^+^|-field, (**c**) |B_1_^−^|-field, and (**d**) |E|-field.

**Figure 3:**
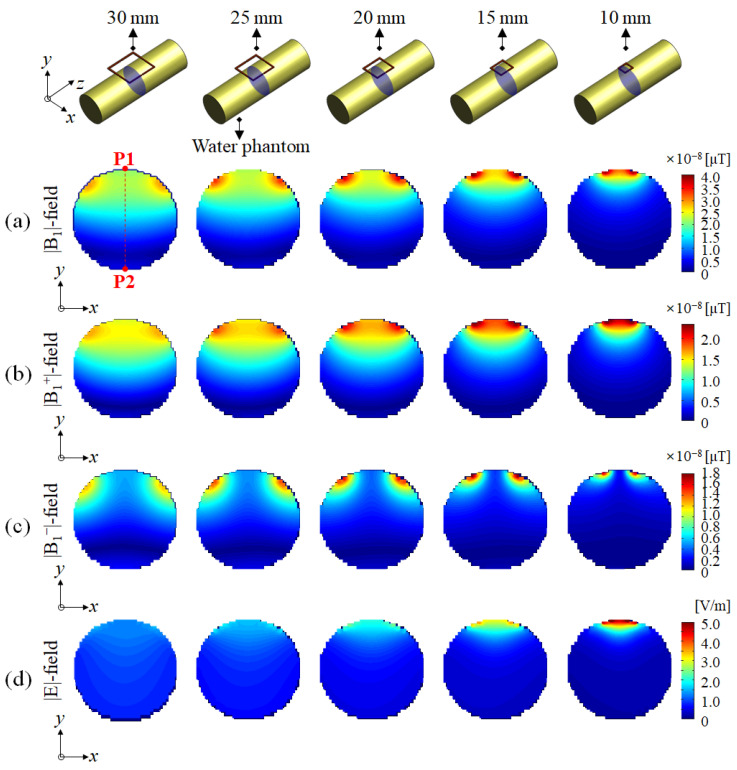
EM-field simulation results using water phantom in the axial slice (*x*-*y* plane): (**a**) |B_1_|-field, (**b**) |B_1_^+^|-field, (**c**) |B_1_^−^|-field, and (**d**) |E|-field.

The signal depth of the |B_1_|-field was compared using a slice profile in the direction of RF penetration from P1 to P2 in the A–P (anterior to posterior) direction along the *y*-axis, from the center of the single-channel surface RF coil, as shown in Figure 2 and Figure 3. Meanwhile, the SAR-map on the basis of the |E|-field concentration was validated for determining the RF safety of a single-channel surface RF coil. In addition, for a more quantitative comparison, the maximum, mean, and standard deviation (STD) values were measured from the SAR-map results.

## 3. Results and Discussion

EM-field distributions using an oil phantom were calculated using the |B_1_|-, |B_1_^+^|-, |B_1_^−^|-, and |E|-field and were compared according to changes in the size of the single-channel surface RF coil, as shown in Figure 2. Typical surface RF coils exhibited strong signal sensitivity at the center of the RF coil and formed a semicircular |B_1_|-field distribution, where relatively low signal sensitivity was observed; however, the signal sensitivity and |B_1_|-field distribution changed as the diameter increased for the single-channel surface RF coils.

Figure 2 shows EM-field distributions in the axial slice (*x*–*y* plane) using an oil phantom with cylindrical geometry. In Figure 2a, the |B_1_|-field distributions of the single-channel surface RF coil with diameters of 10 mm and 15 mm each appeared as typical semicircles in the oil phantom. On the other hand, in the case of a single-channel surface RF coil with a diameter of 20 mm, the difference in |B_1_|-field sensitivity between the center of the coil and the periphery regions was markedly reduced. For the |B_1_|-field with diameters of 25 mm and 30 mm, a lower signal sensitivity was observed at the center of the single-channel surface RF coil than in the periphery region. In Figure 2b, the |B_1_^+^|-field distribution tended to be similar to that of the |B_1_|-field distribution, whereas the |B_1_^−^|-field distribution in Figure 2c showed a different tendency from that of the |B_1_^+^|-field distribution. According to the reciprocity theorem [70], the surface RF coils had an equal distribution as that of the |B_1_^+^|-field for RF transmission and the |B_1_^−^|-field for RF reception. However, as the magnetic field strength increased, the reciprocity theorem was not established due to inhomogeneity between RF transmission and RF reception. Figure 2d shows the |E|-field concentration of a single-channel surface RF coil. The maximum |E|-field value was observed with a diameter of 10 mm, and the |E|-field distribution relatively decreased as the diameter of the single-channel surface RF coils increased. According to the EM-field simulation results shown in Figure 2, the farther the distance from the single-channel surface RF coil along P1–P2, a more linear reduction field pattern was observed in the EM field. As shown in Figure 2, a single-channel surface RF coil consisting of a diameter of 20 mm provided optimal |B|-field distribution. On the other hand, surface RF coils with a diameter of more than 25 mm increased the signal sensitivity of the |B|-field in the periphery region rather than in the center of the coil.

EM-field simulation results of the water phantom using distilled water modeled to the same size as the oil phantom were calculated as shown in Figure 3. EM-field distributions using the water phantom were calculated using the |B_1_|-, |B_1_^+^|-, |B_1_^−^|-, and |E|-fields and were compared in the same way as the EM-field simulation results using the oil phantom in Figure 2.

In the EM-field simulation result using the water phantom, as shown in Figure 3, a deeper signal depth could be observed in the |B_1_|- and |B_1_^+^|-fields compared to the result using the oil phantom (in Figure 2). This was due to the characteristics of the water phantom using distilled water that had higher dielectric properties than the oil phantom. The simulation results using the water phantom showed that the EM-field was distorted along the *y*-direction (near the P2 point), but it was not found in the EM-field simulation result using the oil phantom. It was possible to observe an EM-field distribution in which the signal strength increases in the opposite direction where the single-channel surface RF coil was located.

**Figure 4:**
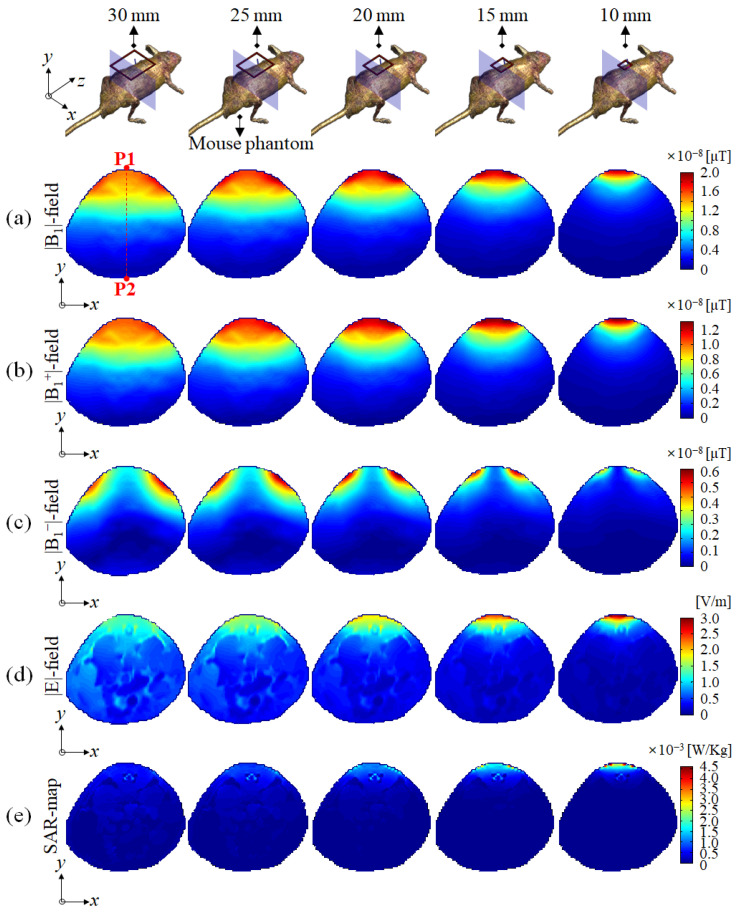
EM-field simulation results using mouse phantom in the axial slice (*x*–*y* plane): (**a**) |B_1_|-field, (**b**) |B_1_^+^|-field, (**c**) |B_1_^−^|-field, (**d**) |E|-field, and (**e**) SAR-map.

In Figure 4, EM-field distributions using a mouse phantom were verified for the |B_1_|-field, |B_1_^+^|-field, |B_1_^−^|-field, |E|-field, and SAR-map, depending on the change in the size of the single-channel surface RF coil. Figure 4 shows that the results of using a mouse phantom were similar to the results obtained using an oil phantom and water phantom, as shown in Figure 2 and Figure 3. However, due to the narrowed dorsal shape of the mouse phantom, high signal sensitivity patterns close to the microstrip line of the single-channel surface RF coil were not observed, which contrasted with the patterns shown in the oil phantom and water phantom results. In addition, the distorted EM-field distribution with reversed signal intensity near the P2 point observed in the water phantom result was equally observed in the |B_1_|-, |B_1_^+^|-, and |B_1_^−^|-field distribution.

In Figure 4a, typical |B_1_|-field distribution was observed in a single-channel surface RF coil with a diameter of 20 mm, but a single-channel surface RF coil with a diameter of more than 25 mm was observed to flatten the |B_1_|-field distribution. For the |B_1_^+^|-field distribution, shown in Figure 4b, we verified field patterns that were similar to those of the |B_1_|-field distribution, as shown in Figure 4a. The |B_1_^−^|-field distribution shown in Figure 4c produced a non-linear field pattern in which the |B_1_^−^|-field distribution increased at a certain point without linearly decreasing. Figure 4d shows the |E|-field distribution using a mouse phantom as the single-channel surface RF coil diameter increased. The |E|-field concentration generated a maximum value near the single-channel surface RF coil, and penetration into the mouse phantom was observed as the diameter of the single-channel surface RF coil increased. Meanwhile, unlike that shown in Figure 2d, the penetration depth of the |E|-field was observed to deepen due to the dielectric properties of the mouse phantom consisting of various tissues. In terms of RF safety, the SAR-map results in Figure 4e showed a similar tendency as the |E|-field results shown in Figure 4d. The results of the single-channel surface RF coil with a diameter of 10 mm showed that the SAR field was concentrated close to the single-channel surface RF coil. Meanwhile, the SAR distribution penetrated the mouse model due to an increase in the diameter of the single-channel surface RF coil.

Table 1 summarizes the max values, mean values, and STD values obtained from the SAR-map distribution in Figure 4. The maximum SAR value was highest at 4.661 × 10^−3^ W/Kg on a single-channel surface RF coil with a diameter of 10 mm and decreased as the diameter of the single-channel surface RF coil increased. The maximum value of the SAR-map was reduced by approximately 25% when the diameter of the single-channel surface RF coil increased from 10 mm to 15 mm, and by approximately 47% when the diameter increased from 15 mm to 20 mm. Increasing the diameter of a single-channel surface RF coil from 20 mm to 25 mm reduced the maximum SAR by 20%, and increasing from 25 mm to 30 mm reduced the maximum SAR by 16%. The SAR values varied rapidly with a single-channel surface RF coil with a diameter of 20 mm, and there was a similar tendency in the mean value and STD distributions.

**Figure 5:**
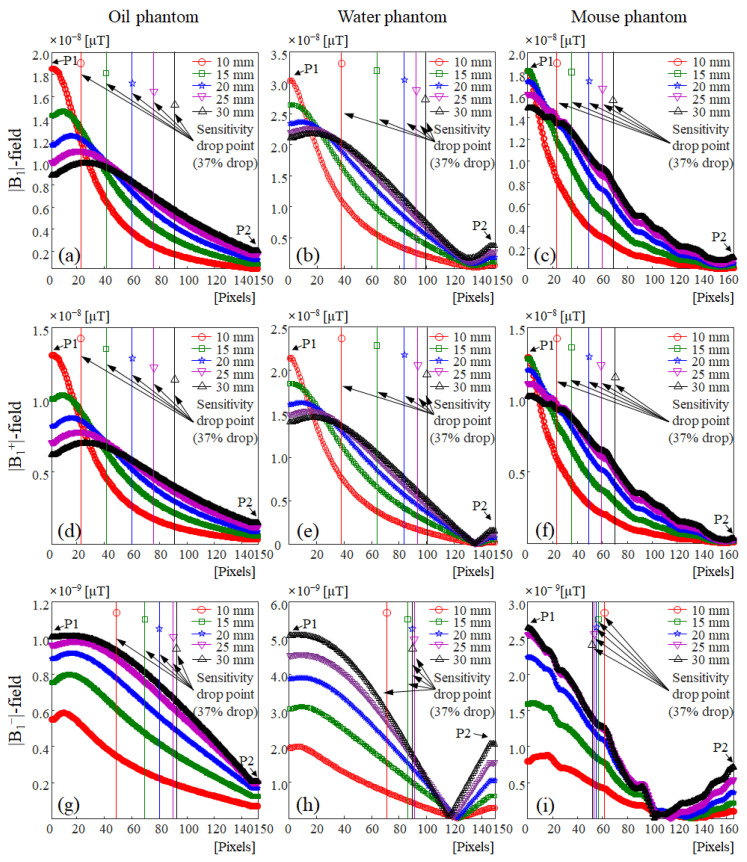
Center slice profile (from P1–P2) of |B_1_|-, |B_1_^+^|-, and |B_1_^−^|-field using oil phantom, water phantom, and mouse phantom: (**a**) center slice profile of |B_1_|-field using oil phantom, (**b**) center slice profile of |B_1_|-field using water phantom, (**c**) center slice profile of |B_1_|-field using mouse phantom, (**d**) center slice profile of |B_1_^+^|-field using oil phantom, (**e**) center slice profile of |B_1_^+^|-field using water phantom, (**f**) center slice profile of |B_1_^+^|-field using mouse phantom, (**g**) center slice profile of |B_1_^−^|-field using oil phantom, (**h**) center slice profile of |B_1_^−^|-field using water phantom, (**i**) center slice profile of |B_1_^−^|-field using mouse phantom.

In Figure 5, the signal depth according to the size of the single-channel surface RF coil was compared with a resolution of 0.2 mm per point in the *y*-axis direction using a slice profile between the P1 and P2 points, as shown in Figure 2, Figure 3 and Figure 4. As can be seen from the simulation results using the oil phantom in Figure 5a,d,g, |B_1_|- and |B_1_^+^|-fields indicated similar slice profiles, except for differences in the signal sensitivity. The signal sensitivity of the |B_1_|- and |B_1_^+^|-fields tended to decrease with the increasing diameter of the single-channel surface RF coil, and in the |B_1_^−^|-field distribution, the signal sensitivity tended to increase with the increasing diameter of the single-channel surface RF coil. The |B_1_^−^|-field results in Figure 5g showed an opposite tendency from the |B_1_|- and |B_1_^+^|-field distributions. This tendency was similar in the EM simulation of the water phantom (shown in Figure 5b,e,h) and mouse phantom (shown in Figure 5c,f,i), but unlike EM simulation with the oil phantom, signal sensitivity was reversed at approximately 23.6 mm (at 118 points) from a single-channel surface RF coil in the |B_1_^−^|-field distribution of the water phantom (in Figure 5h). This signal reversal was also observed in the |B_1_^−^|-field distribution of the mouse phantom (in Figure 5i), with the signal sensitivity reversed at approximately 21 mm (105) on the single-channel surface RF coil. The |B_1_^−^|-field sensitivity reversed in this way was caused by the inhomogeneity of the main magnetic field and the complex dielectric properties of the mouse phantom in UHF MRI [75,76].

The signal depth, an important measure for evaluating the performance of a single channel surface coil, was defined as the point where the coil’s sensitivity drops to 37% of that at the center of the coil [73,74]. To evaluate the signal depth of the central slice profile in the |B_1_|- and |B_1_^+^|-fields, the point at which the signal intensity decreased by 37% based on the field sensitivity value of the point P1 (in Table 2) was measured and shown in Table 3. In the results of the oil phantom and mouse phantom in Figure 5 and Table 3, for single-channel surface RF coils with a size of 10 mm and 15 mm, the signal depth of |B_1_|- and |B_1_^+^|-fields were less than 10 mm and sufficient signal depths were not provided. However, in the results of the |B_1_|- and |B_1_^+^|-fields, a single-channel surface RF coil with a diameter of 20 mm provided a sufficient signal depth of more than 10 mm. Exceptionally, the water phantom results showed that a sufficient signal depth of more than 10 mm was provided in a single-channel surface RF coil with a diameter of 15 mm. The results of the |B_1_|- and |B_1_^+^|-fields of the water phantom showed that the signal depth increased rapidly as the diameter of the single-channel surface RF coil decreased compared to the oil phantom result. It was confirmed that there was a high similarity between the water phantom result and the mouse phantom result in the |B_1_^−^|-field distribution. In |B_1_^−^|-field results (shown in Table 3), the difference in signal depth according to the size change of the single-channel surface RF coil was measured to be up to 3.8 mm with the water phantom and up to 1.6 mm with the mouse phantom.

The brain of the mouse phantom used in the EM-field simulation had a height of 8 mm, and the signal depth had to be 10 mm or more to obtain an image of the entire mouse brain region, considering the skull and skin. In addition, the signal depth had to be at least 10 mm to obtain spinal cord images of the mouse’s body. In other words, in order to obtain mouse brain and spinal code images in preclinical MRI experiments using mice, a single-channel surface RF coil with a diameter of at least 20 mm had to be used.

The effectiveness of the proposed 20 mm diameter surface RF coil in mouse experiments using preclinical 9.4 T MRI could be confirmed not only in axial slices but also in the results of the sagittal slice (in Figures S1–S3) and coronal slice (in Figures S4–S6). As a result of the distribution of EM-fields using the oil phantom (in Figure S1), water phantom (in Figure S2), and mouse phantom (in Figure S3) in the sagittal slice, it was confirmed that the same distribution as the axial slice appeared. In addition, according to the results of the EM-fields using the oil phantom (in Figure S4), water phantom (in Figure S5), and mouse phantom (in Figure S6) in the coronal slice, it was confirmed that the single-channel surface RF coil with a diameter of 20 mm is the minimum usage criteria.

In this paper, according to the results while using the oil phantom, water phantom, and mouse phantom, a single-channel surface RF coil consisting of a 20 mm diameter provided an optimal |B_1_|-field distribution and confirmed that single-channel surface RF coils with a diameter of more than 25 mm could not provide a typical |B_1_|-field distribution within the guidelines for RF safety. To preserve the characteristics of typical single-channel surface RF coils and ensure that the RF signal is correctly applied to the target point, we proposed using a single-channel coil with a diameter of 20 mm for 9.4 T MRI.

To discuss the results of this study, we proposed a single-channel RF coil with a diameter of 20 mm optimized for small mice at 9.4 T MRI. The proposed 20mm diameter single-channel RF coil provided signal depth of RF coil suitable for mouse experiments in the 9.4 T MRI study and confirmed the RF safety of experimental animals.

Although the proposed 20 mm diameter single-channel RF coil was expected to be readily applicable to preclinical 9.4 T MRI studies, this study was conducted with two limitations.

First, comparative studies on MRIs of various magnetic field intensities had not been conducted. The EM field characteristics and SAR distribution of the RF coil change due to the decrease in the wavelength of the RF frequency depending on the strength of the magnetic field. In this paper, we proposed a single-channel RF coil optimized only for 9.4 T MRI but failed to compare the electromagnetic field and RF safety of a single-channel RF coil in preclinical MRIs with different magnetic field intensities. In the following study, a comparative analysis of preclinical MRI of various magnetic field strengths such as 3.0 T, 7.0 T, 11.4 T, and 21.0 T is required [44,77,78,79,80].

Second, the RF coil size required in MRI experiments is typically defined according to the type and size of the target animal and the image acquisition region. The EM-field simulations using various experimental animal models are required to accurately assess the performance of the single-channel surface RF coil in preclinical MRI. Therefore, comparative studies on experimental animals of various sizes and types are essential. The EM-field simulation program used in this study can apply various animal phantoms provided by the IT’IS Foundation, enabling comparative studies on animals of various sizes and types.

Experimental animal models for EM-field simulations provided by the IT’IS Foundation provide 17 animal phantoms consisting of a total of 9 species of animals, as shown in Table S1. However, it takes a lot of time to conduct comparative research on various species of animals, and it is difficult to compare all of them within a limited page-length manuscript. Therefore, in this study, size optimization was performed according to the size of the single-channel surface RF coil using only mice, which are the most frequently used in animal experiments [81,82]. In recent research trends using experimental animals (especially in Asia, the European Union, and the United Kingdom), experiments using mice and rats account for 70–80% of the total animal experiments, of which the utilization rate of mice is overwhelmingly high at 60–70% [83].

To solve these two limitations, a comparative study using various main magnetic field strengths and various animal phantoms will be performed. In addition, based on the proposed single-channel surface RF coil with a diameter of 20 mm in this study, we are planning to extend the suggested single-channel surface RF coil to a multi-channel RF coil and are also planning an optimized diameter to provide RF safety of experimental animals.

## 4. Conclusions

In preclinical MRI, single-channel surface RF coils have been used in the Tx/Rx mode for small animal studies using rat or mouse models due to their high signal sensitivity. However, as the main magnetic field strength increases, single-channel surface RF coils of optimized size have been used without performing quantitative analysis or following the guidelines of RF safety. Therefore, it is essential to define a single-channel surface RF coil that has been optimized according to the main magnetic field strength and with consideration for the signal depth to ensure that the RF signal is correctly applied to the target image acquisition point within the RF safety guidelines.

From the results of this study, the sensitivity and signal depth of |B_1_|-, |B_1_^+^|-, and |B_1_^−^|-field, including RF safety, were verified by using numerical calculations for preclinical MRI. A single-channel surface RF coil of 10 mm and 15 mm each provided high |B_1_|-field sensitivity, and the small size of the single-channel surface RF coil caused an |E|-field concentration, thus resulting in an increase in the SAR value. The use of single-channel surface RF coils with diameters of 10 mm and 15 mm in a preclinical MRI doubled the SAR compared to the use of single-channel surface RF coils with a diameter of 20 mm at a location close to a single-channel surface RF coil, thus resulting in many drawbacks in SAR safety for experiments using small animals. In preclinical MRI, single-channel surface RF coil acquires images as close as possible to the target small animal, and an increase in the maximum SAR due to the |E|-field concentration lead to a burn injury or changes in the biological properties of the small animal. 

Considering RF safety for small animals, the optimal single-channel surface RF coil size should be at least 20 mm, and it is recommended to use a 20 mm single-channel surface RF coil while considering high signal sensitivity, signal depth, and RF safety. In this paper, we verified the efficiency and safety of the single-channel surface RF coil with a diameter of 20 mm for mouse experiments in 9.4 T preclinical MRI. The proposed 20 mm diameter single-channel surface RF coil showed excellent performance in terms of |B_1_|-field signal depth and SAR, which could be utilized to improve the quality of small animal images for preclinical MRI. 

In addition, we also expect to be able to conduct research on RF coils that are suitable for more diverse purposes using methods considering the signal depth and SAR analysis for determining optimized single-channel surface RF coils for preclinical MRI studies. In addition, the proposed 20 mm diameter single-channel surface RF coil can be easily applied to multi-channel RF coils in preclinical 9.4 T MRI systems and will provide high |B_1_|-field sensitivity with sufficient signal depth, and will also satisfy SAR safety of experimental mice.

## Figures and Tables

**Table 1:** SAR values according to the change in size of single-channel surface RF coils using a mouse phantom.

SAR [× 10^−^^3^ W/Kg]	30 mm	25 mm	20 mm	15 mm	10 mm
Max values	1.251	1.488	1.853	3.496	4.661
Mean values	0.163	0.154	0.139	0.117	0.082
STD	0.164	0.206	0.257	0.312	0.337

**Table 2:** Field sensitivity at P1 point according to the change in size of single-channel surface RF coils using oil phantom, water phantom, and mouse phantom.

Field Sensitivity at P1 Point [× 10^−^^8^ μT]		B_1_-Field	B_1_^+^-Field	B_1_^−^-Field
Oil phantom	30 mm	0.885	0.618	0.100
	25 mm	1.002	0.702	0.096
	20 mm	1.159	0.815	0.088
	15 mm	1.424	1.004	0.075
	10 mm	1.849	1.306	0.055
Water phantom	30 mm	2.108	1.401	0.507
	25 mm	2.205	1.492	0.452
	20 mm	2.341	1.610	0.387
	15 mm	2.647	1.847	0.304
	10 mm	3.043	2.142	0.196
Mouse phantom	30 mm	1.488	1.019	0.263
	25 mm	1.160	1.110	0.255
	20 mm	1.728	1.201	0.223
	15 mm	1.824	1.280	0.159
	10 mm	1.825	1.288	0.079

**Table 3:** The point where the sensitivity drops to 37% according to the change in size of single-channel surface RF coils using oil phantom, water phantom, and mouse phantom.

Sensitivity 37% Drop Point [Pixels]		B_1_-Field	B_1_^+^-Field	B_1_^−^-Field
Oil phantom	30 mm	92 (18.4 mm)	92 (18.4 mm)	92 (18.4 mm)
	25 mm	77 (15.4 mm)	77 (15.4 mm)	89 (17.8 mm)
	20 mm	60 (12.0 mm)	60 (12.0 mm)	80 (16.0 mm)
	15 mm	41 (8.2 mm)	41 (8.2 mm)	68 (13.6 mm)
	10 mm	23 (4.6 mm)	22 (4.4 mm)	47 (9.4 mm)
Water phantom	30 mm	99 (19.8 mm)	100 (20.0 mm)	89 (17.8 mm)
	25 mm	93 (18.6 mm)	94 (18.8 mm)	91 (18.2 mm)
	20 mm	84 (16.8 mm)	84 (16.8 mm)	89 (17.8 mm)
	15 mm	64 (12.8 mm)	64 (12.8 mm)	86 (17.2 mm)
	10 mm	38 (7.6 mm)	38 (7.6 mm)	72 (14.4 mm)
Mouse phantom	30 mm	68 (13.6 mm)	71 (14.2 mm)	53 (10.6 mm)
	25 mm	60 (12.0 mm)	59 (11.8 mm)	54 (10.8 mm)
	20 mm	50 (10.0 mm)	50 (10.0 mm)	55 (11.0 mm)
	15 mm	35 (7.0 mm)	35 (7.0 mm)	56 (11.2 mm)
	10 mm	23 (4.6 mm)	23 (4.6 mm)	61 (12.2 mm)

## Data Availability

Not applicable.

## Supplementary Materials

The following supporting information can be downloaded at: https://www.mdpi.com/article/10.3390/s22114274/s1, Figure S1. EM-field simulation results using oil phantom in the sagittal slice (*y*–*z* plane): (a) |B_1_|-field, (b) |B_1_^+^|-field, (c) |B_1_^−^|-field, and (d) |E|-field; Figure S2. EM-field simulation results using water phantom in the sagittal slice (*y*–*z* plane): (a) |B_1_|-field, (b) |B_1_^+^|-field, (c) |B_1_^−^|-field, and (d) |E|-field; Figure S3. EM-field simulation results using mouse phantom in the sagittal slice (*y*–*z* plane): (a) |B_1_|-field, (b) |B_1_^+^|-field, (c) |B_1_^−^|-field, (d) |E|-field, and (e) SAR-map; Figure S4. EM-field simulation results using oil phantom in the coronal slice (*x*–*z* plane): (a) |B_1_|-field, (b) |B_1_^+^|-field, (c) |B_1_^−^|-field, and (d) |E|-field; Figure S5. EM-field simulation results using water phantom in the coronal slice (*x*–*z* plane): (a) |B_1_|-field, (b) |B_1_^+^|-field, (c) |B_1_^−^|-field, and (d) |E|-field; Figure S6. EM-field simulation results using mouse phantom in the coronal slice (*x*–*z* plane): (a) |B_1_|-field, (b) |B_1_^+^|-field, (c) |B_1_^−^|-field, (d) |E|-field, and (e) SAR-map. Table S1. Types of experimental animal phantom provided by IT’IS Foundation for EM-field simulation.
