# Supplementary material for: Simulation Study of Radio Frequency Safety and the Optimal Size of a Single-Channel Surface Radio Frequency Coil for Mice at 9.4 T Magnetic Resonance Imaging
Source: Sensors (Basel). 2022 Jun 3;22(11):4274. doi: 10.3390/s22114274 (PMC9185248; doi:10.3390/s22114274)
Supplement: Supplementary file 1 [file sensors-22-04274-s001.zip › sensors-1715970-supplementary.pdf]

sensors

MDPI

Supplementary Materials

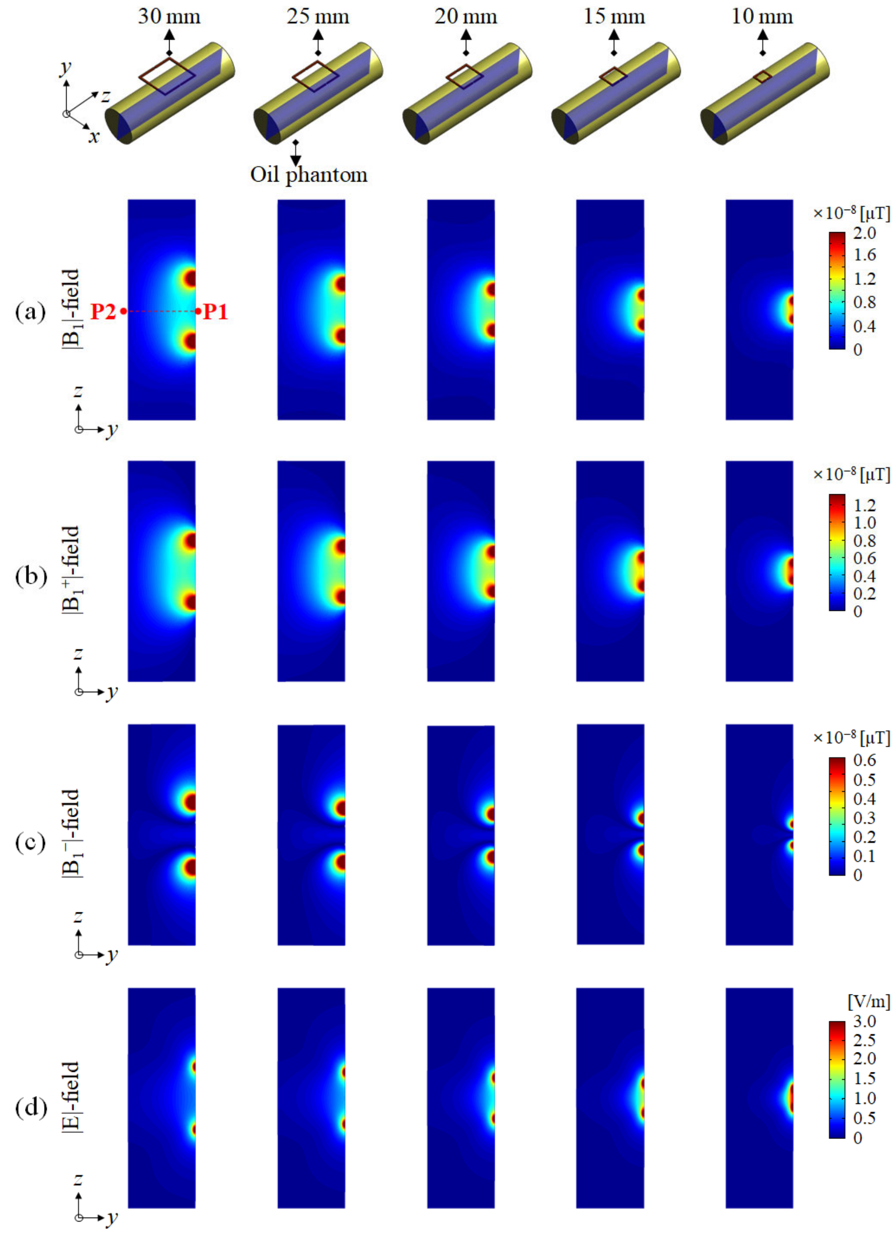

Figure S1. EM-field simulation results using oil phantom in the sagittal slice (*y*-*z* plane): (**a**) $|B_1|$-field, (**b**) $|B_1^+|$-field, (**c**) $|B_1^-|$-field, and (**d**) $|E|$-field.

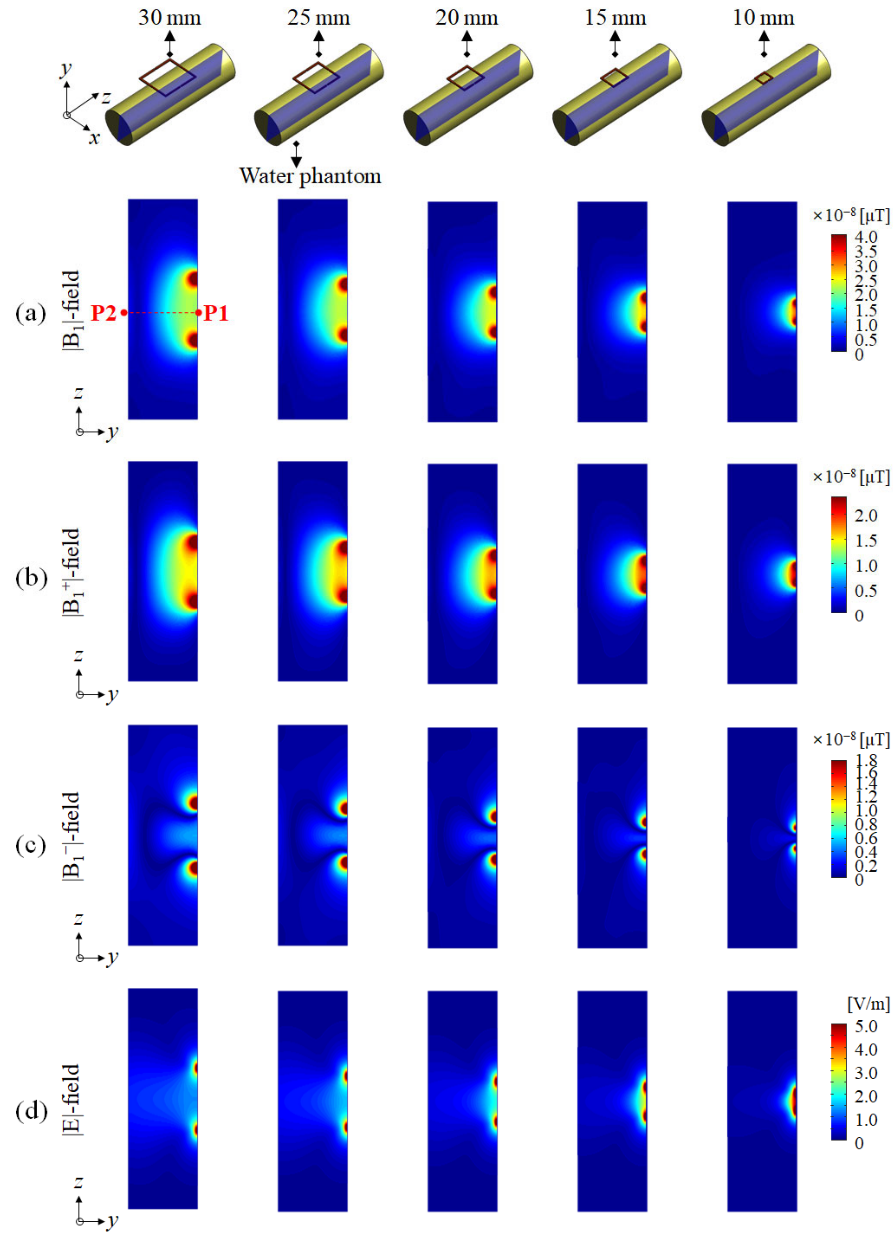

Figure S2. EM-field simulation results using water phantom in the sagittal slice (*y*-*z* plane): (**a**) $|B_1|$-field, (**b**) $|B_1^+|$-field, (**c**) $|B_1^-|$-field, and (**d**) $|E|$-field.

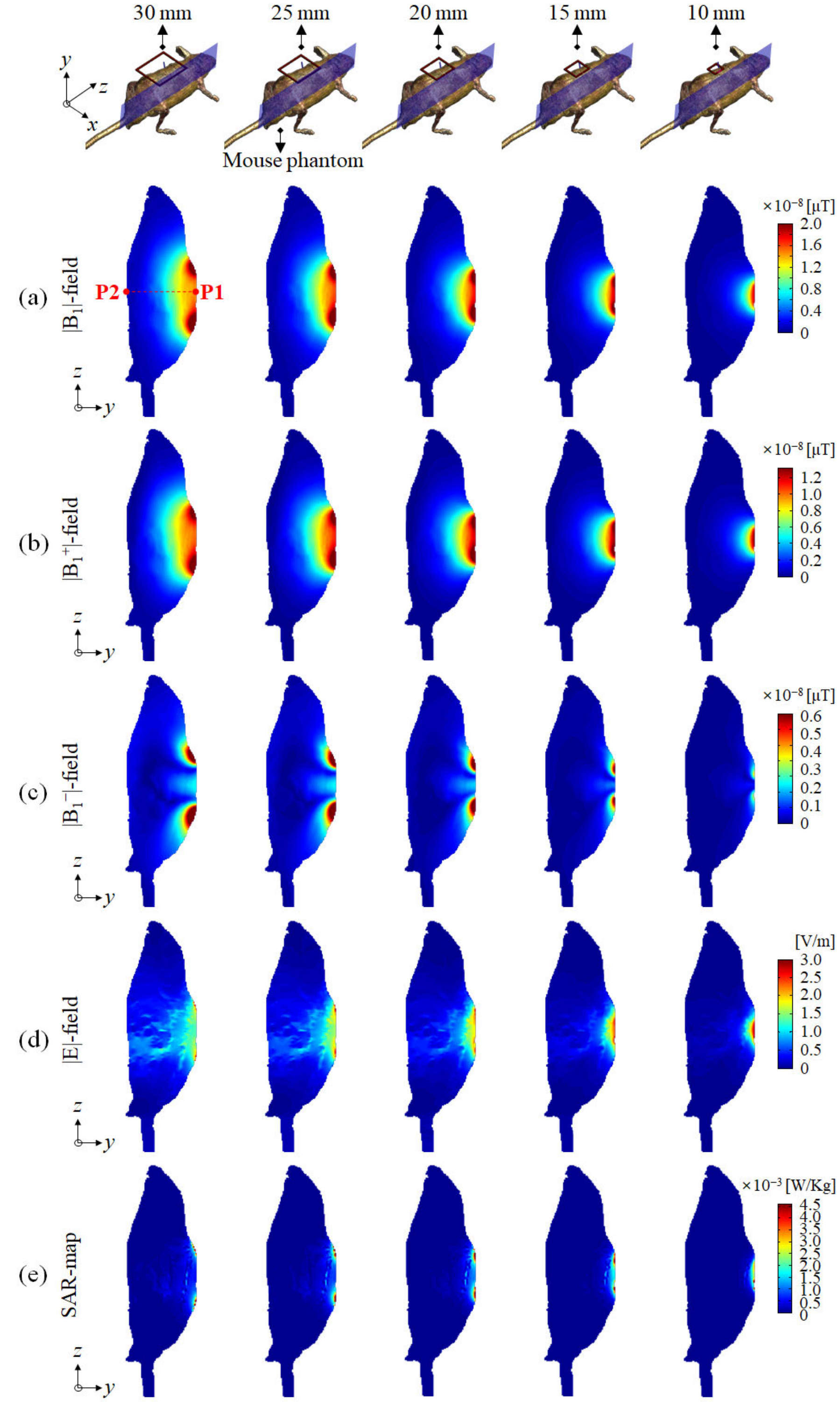

Figure S3. EM-field simulation results using mouse phantom in the sagittal slice (*y*-*z* plane): (**a**) $|B_1|$-field, (**b**) $|B_1^+|$-field, (**c**) $|B_1^-|$-field, (**d**) $|E|$-field, and (**e**) SAR-map.

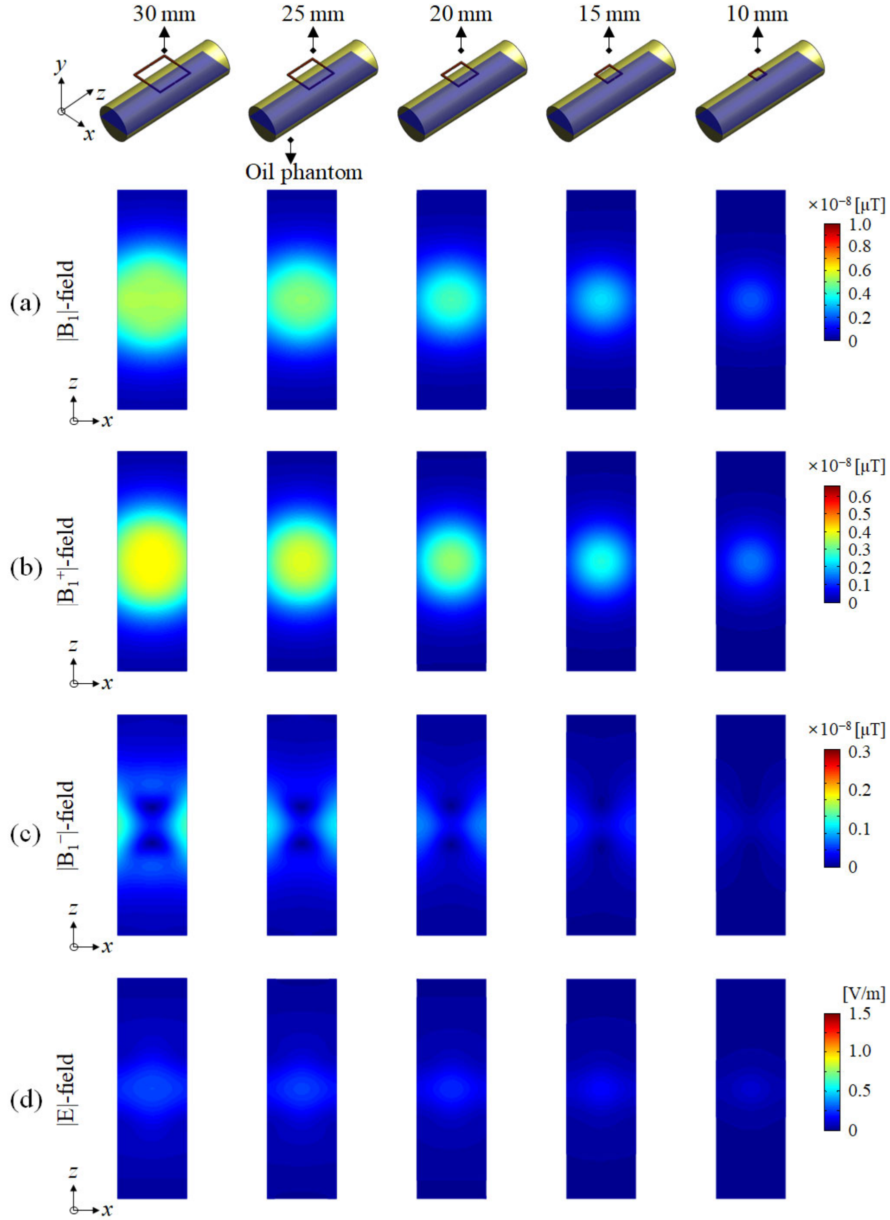

Figure S4. EM-field simulation results using oil phantom in the coronal slice (*x*-*z* plane): (a) $|B_1|$-field, (**b**) $|B_1^+|$-field, (**c**) $|B_1^-|$-field, and (**d**) $|E|$-field.

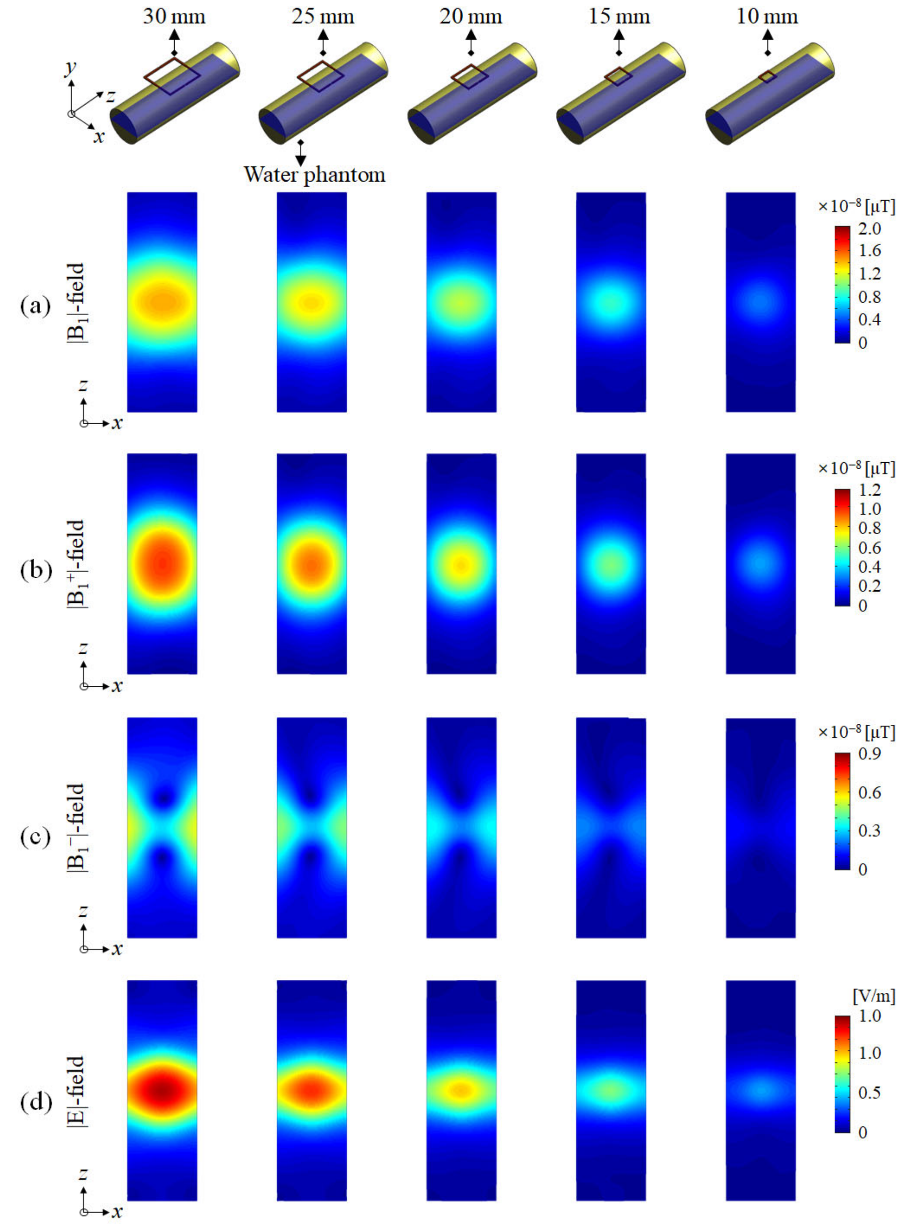

Figure S5. EM-field simulation results using water phantom in the coronal slice (*x*-*z* plane): (**a**) $|B_1|$-field, (**b**) $|B_1^+|$-field, (**c**) $|B_1^-|$-field, and (**d**) $|E|$-field.

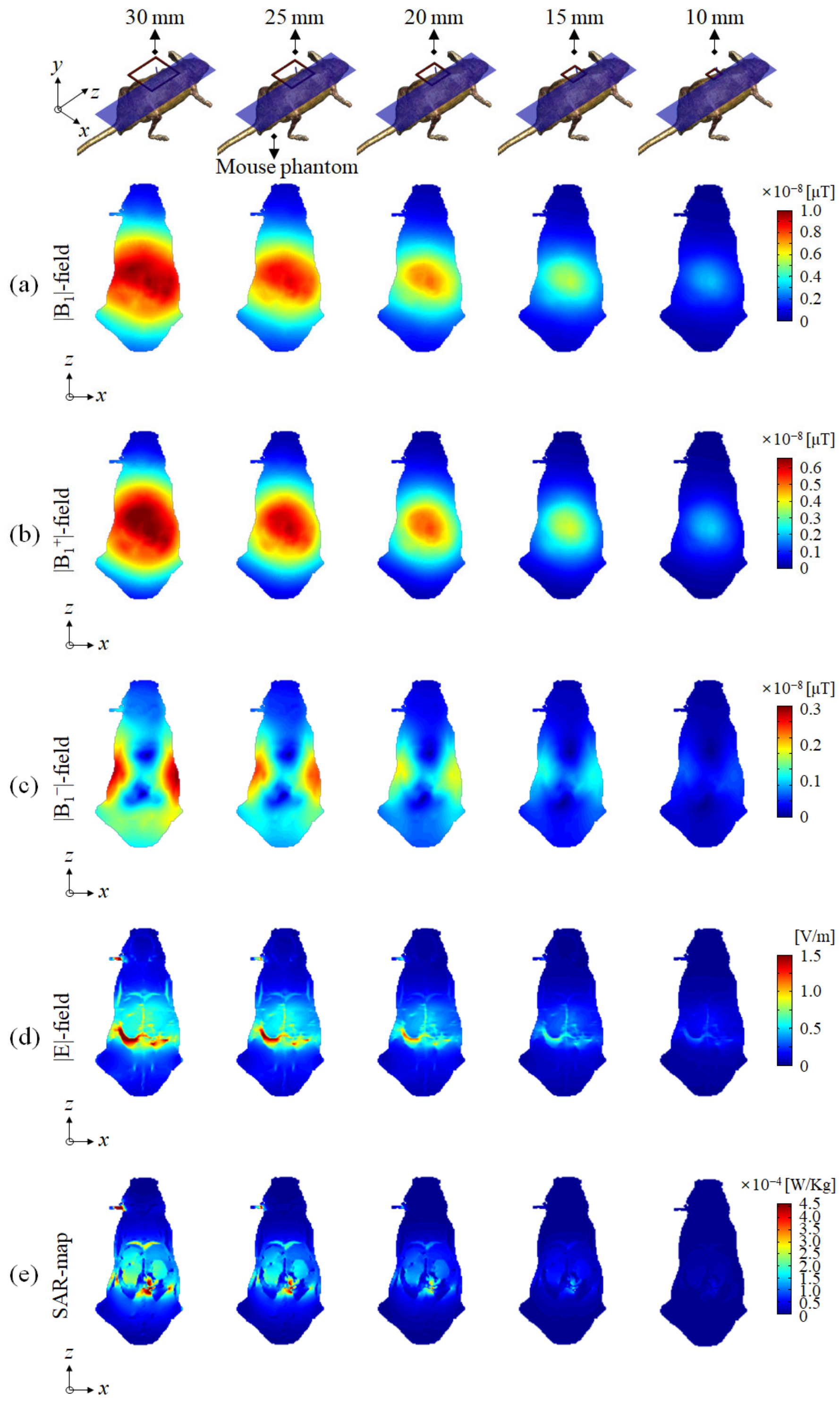

Figure S6. EM-field simulation results using mouse phantom in the coronal slice (*x*-*z* plane): (**a**) $|B_1|$-field, (**b**) $|B_1^+|$-field, (**c**) $|B_1^-|$-field, (**d**) $|E|$-field, and (**e**) SAR-map.

Table S1. Types of experimental animal phantom provided by IT'IS Foundation for EM-field simulation.

Name	Type	Length without Tail [mm]	Weight [g]	Number of Tissues
"Miss Able" Female Monkey	Rhesus macaque	740	4900	356
Male Pig	Domestic Pig	977	35000	103
NeuroRat (male)	Dark Agouti	150	150	179
Big Male Rat	Sprague Dawley	260	567	51
Small Male Rat	Sprague Dawley	185	198	52
Female Rat with Tumors	Sprague Dawley	225	503	50
Pregnant Rat (female)	Sprague Dawley	170	275	52
Rat Pup (undefined)	Sprague Dawley	93 (with tail)	14.3	49
Male PIM1 Mouse	PIM1	98	44.7	49
Male OF1 Mouse	OF1	95	35.5	50
Female OF1 Mouse	OF1	78	17.3	48
Pregnant Mouse (female)	C57BL/6N	160	38	68 + 10 embryos
"Diggy" Male Nude Normal Mouse	Nude Normal	86	28	43
Pregnant Mouse (female)	B6C3F1	72	28.7	46
3 Week Male Mouse	B6C3F1	70	12.3	67
12 Week Female Mouse	B6C3F1	80	22.3	68
12 Week Male Mouse	B6C3F1	90	27.4	67
